# MemoBox: A mechanical follow-the-leader system for minimally invasive surgery

**DOI:** 10.3389/fmedt.2022.938643

**Published:** 2022-09-13

**Authors:** Costanza Culmone, David J. Jager, Paul Breedveld

**Affiliations:** ^1^ BITE Group, Department of Biomechanical Engineering, Faculty of Mechanical, Maritime, and Materials Engineering, Delft University of Technology, Delft, Netherlands; ^2^ Department of Electronic and Mechanical Support Division, Delft University of Technology, Delft, Netherlands

**Keywords:** follow-the-leader, minimally invasive surgery, path follow, medical device, snake-like instruments

## Abstract

With the increase in Natural Orifice Transluminal Endoscopic Surgery procedures, there is an increasing demand for surgical instruments with additional degrees of freedom, able to travel along tortuous pathways and guarantee dexterity and high accuracy without compromising the surrounding environment. The implementation of follow-the-leader motion in surgical instruments allows propagating the decided shape through its body and moving through curved paths avoiding sensitive areas. Due to the limited operational area and therefore the instrument size, the steerable shaft of these instruments is usually driven by cables that are externally actuated. However, a large number of degrees of freedom requires a great number of actuators, increasing the system complexity. Therefore, our goal was to design a new memory system able to impose a follow-the-leader motion to the steerable shaft of a medical instrument without using actuators. We present a memory mechanism to control and guide the cable displacements of a cable-driven shaft able to move along a multi-curved path. The memory mechanism is based on a programmable physical track with a mechanical interlocking system. The memory system, called MemoBox, was manufactured as a proof-of-concept demonstration model, measuring 70 mm × 64 mm × 6 mm with 11 programmable elements and featuring a minimum resolution of 1 mm. The prototype shows the ability to generate and shift complex 2D pathways in real-time controlled by the user.

## Introduction

Minimally invasive surgery (MIS) aims at reducing the invasiveness of a surgical procedure by using small incisions as the entry port of the human body. By reducing the incision size, the chance of exposure-related infections, pain, and recovery time decrease drastically. A step forward has been made with the introduction of Natural Orifice Transluminal Endoscopic Surgery (NOTES), in which surgeons can operate and enter the human body using natural orifices such as mouth, nose, or anus (1–3). For instance, Endoscopic Endonasal Surgery (EES) is a NOTES procedure in which the nostrils are the entry port to reach and remove tumors at the base of the skull, such as those occurring in the pituitary gland. The pituitary gland is difficult to reach because the nostrils create a narrow passage that limits the maneuverability of the tools. Therefore, by using conventional straight and rigid instruments, some tumors cannot be reached or removed entirely, and patients require further treatment (4, 5). Instruments such as flexible endoscopes or catheters usually have a passively flexible shaft, and only in some of them, the end-segment can be actively controlled and articulated. These instruments usually need support from the surrounding environments that constrain and guide them through organs such as the intestines or the blood vessels. However, soft tissues and delicate anatomies, such as those in the skull base and around the pituitary gland, cannot provide enough support for such instruments, leading to the need for having instruments capable of self-support and self-guidance.

In 1999, Choset and Henning minted the new term “Follow-the-Leader” (FTL), also known as path following, to refer to a new kind of motion behavior of segmented snake-like robots (6). In FTL-motion, the user steers the head (the most distal segment) of the robot. The pose information is stored and passed back to the other segments in order to let them assume the same pose once they have translated to the same location in space. Therefore, the user only controls the position of the first segment, the so-called “leader”. At the same time, the other segments follow the trajectory created by the leader, mimicking the obstacle-avoiding motion of a snake through its environment. The controlled navigation of FTL-instrumentation can be a valuable alternative not only in EES but in many other surgical scenarios. Applications can also be found in interventional bronchoscopy, in which a steerable bronchoscope is inserted into the bronchi to perform diagnostic biopsies. Bronchi branches are delicate and thin, and when the target lesion is located in a peripheric area of the lungs, the diagnostic sensitivity, which is the percentage of successfully diagnosed lesions, is very low (<25%) (7). FTL-instruments could bring bronchoscopy to a higher level and help the surgeon to navigate through peripheric bronchi and increase the diagnostic sensitivity. Another possible application is in the trans-catheter replacement of cardiac valves such as the aortic valve (8) or mitral valve (9). During these procedures, the femoral artery for the aortic valve, or the femoral vein for the mitral valve, are used to insert the catheter and bring the new valve to the heart. Especially in the mitral valve replacement, the catheter needs to navigate through the beating heart to position the new valve correctly. During navigation, FTL-instruments could help the surgeon to precisely control the motion for catheter insertion and retraction.

## State-of-the-art

### Snake-like surgical robots

**Figure 1:**
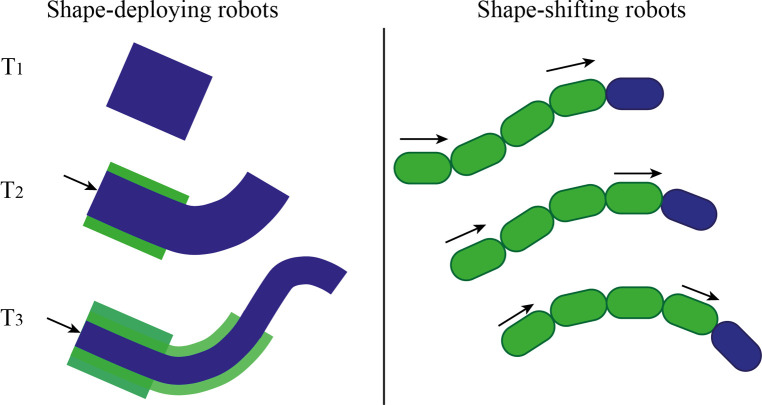
Categorization of FTL surgical robots with extrinsic actuators: shape-deploying (left) and shape-shifting robots (right). Blue indicates the leader segment and green the follower segment(s). T1, T2, and T3 indicate three different phases of the shape propagation.

FTL-motion has been implemented into a number of surgical instruments. Snake-like robots based on a hyper-redundant structure have each rigid segment connected to the adjacent segment(s) by means of rigid joints. Those rigid joints are usually driven by individual embedded actuators (intrinsic). Having individual actuators allows direct control over the segment; however, miniaturization is challenging (10, 11). Therefore, possible applications are limited to procedures such as colonoscopy, in which the diameter of the instruments can be larger than 10 mm (12–14). Miniaturization is instead possible with actuators placed outside the main body of the instrument (extrinsic), leading to fewer spatial limitations (15, 16), less issues with sterilization (17), and possible low-cost disposable use (18, 19). Surgical robots can also be categorized into two main groups considering the shape propagation method: shape-deploying and shape-shifting (20, 21) (Figure 1).

Shape deploying robots mainly comprise telescopic robots and alternating robots. Telescopic robots are usually concentric tube continuum robots based on pre-curved elastic components concentrically nested into each other. Motors are placed outside the robot and enable sliding and rotation of the tubes over one another. Due to the difference in stiffness of the concentric tubes, the robot can create various paths (22, 23). These robots can achieve FTL-motion by controlling the actuation mechanism; however, FTL-motion is limited to specific paths related to the pre-curves of the elastic tubes, requiring prior planning of the trajectory (24, 25). The research group of Burgner-Kahrs developed a hybrid cable-driven continuum robot in which a telescopic backbone is combined with magnetic spacer disks to control the arc lengths and the curvature of the bending section during the FTL-motion (16, 26). They also present an alternative hybrid continuum robot in which two nested Nitinol tubes, equipped with spacer disks, use a cable-driven actuation method to achieve FTL-motion (27). An evaluation of these two robots showed that they were able to perform specific single and double-curved paths.

Alternating robots are based on actively stiffening their components in an alternating fashion to create the desired path (20). The Highly Articulated Robot Probe (HARP), also known as CardioArm or FlexRobotic System (Medrobotics Corp., Raynham, MA, USA), is based on a friction-based locking system between rigid cylindrical links and spherical joints (28–30). The alternation between a rigid and a limp state of two concentric tubes allows the robot to follow a specific path decided in real-time by the user. However, due to the large dimensions (Ø10 mm in the commercialized version), applications are limited to the colon, rectum, or laryngopharyngeal complex (31, 32).

In shape-shifting robots, the position and steering angle of the first distal segment are actively controlled by the user, whereas the following segments assume the steering angle and position of the segment in front of them as the instrument moves ahead. Usually, surgical robots that belong to this group are made of a large number of segments (hyper-redundant). The segments are steered by cables and individually controlled by electric motors placed at the proximal part of the instrument and therefore separated from the robot's snake-like body (33–35). In these robots, each degree of freedom (DOF) requires a dedicated actuator as transferring back the shape among the follower segments requires simultaneous control of all the segments. The use of a large number of motors leads to a complexity higher than strictly necessary for FTL-motion that, in principle, only needs to actively control the pose of the leader segment and passively transfer it to the follower segments.

### Mechanical shape-shifting devices

A first attempt to avoid the use of electrical actuators in shape-shifting devices is a fully mechanically-controlled and cable-driven instrument developed by Henselmans et al. (36). They developed a master-slave system in which a pre-curved steel rod is read out by the master, which passes the pose information to a Ø5 mm slave shaft that mimics the shape of the steel rod. A second fully mechanically-controlled and cable-driven prototype is the so-called MemoFlex II (21). This device has a snake-like shaft of 16 segments that are steered by steering cables. The steering cables are fixed to control points, and the main body of the device (also called revolver) allows their backward and forward motion. To define the path of the control points, a track ring that contains fixed curved grooves representing pre-defined physical tracks rotates around the revolver and guides the control points. A chassis synchronizes the rotation of the track ring with the forward motion of the snake-like shaft using coupled grooves so that the motion of the shaft will correspond with the pre-defined curvature of the fixed tracks.

Although both these instruments function quite well, they have the drawback of being designed to follow a pre-defined path. By using pre-operative MRI or CT imaging data, the surgeon, determines the path to be followed beforehand without the possibility of changing direction or adjusting the position of the shaft during the procedure, limiting his/her action.

Therefore, in this work, we propose a fully mechanical solution that allows real-time control of FTL shape-shifting devices, such as the one proposed by Helselmans et al. (21). The memory system mechanically memorizes the pose information of the leading segment and propagates the shape to the follower segments. The direct guidance of the user over the leading segment position allows control without *a priori* knowledge of the path. The mechanism can perform a variety of different path shapes, such as single, double, or multi-curved paths. Moreover, the mechanical memory controls all the shaft degrees of freedom (DOF) by using only the input given by the user. Therefore, the system complexity is reduced by the decoupling of the DOF from the number of control actuators used in motorized systems (33–35, 37).

## Concept design

### Cable-driven FTL-propagation

**Figure 2:**
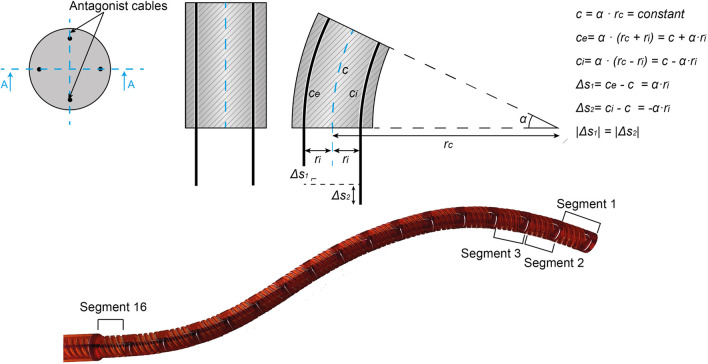
Cable-driven segments. Four-cable control of a steerable segment and an example of a Ø8 mm cable-driven shaft with 16 segments. Segment 1 is the leader segment. Adapted from (21).

Shape-shifting devices are usually based on cable-driven mechanisms that offer the possibility of having a high number of degrees of freedom while keeping the diameter of the shaft small. The minimum number of cables for steering one segment in two DOF is three. The use of four cables, placed in diametrically opposite positions in the shaft cross-section, allows antagonistic movement of the cables, simplifying their control. Figure 2 shows that when a steerable segment is bent to an angle *α* by applying a specific displacement Δs to a steering cable, the antagonistic cable is displaced by the same Δs in the opposite direction. Thus, passing the displacement Δs of the leader segment to the follower segments allows an FTL-motion in a shape-shifting device. Figure 2 shows an example of a multi-steerable shaft with 16 segments. Each segment is controlled by four cables and has two DOF. In an FTL-motion of the shaft, the displacement Δs of leader segment 1 shifts back to the follower segment 2. Then, leader segment 1 will assume a new pose, and the new displacement Δs will be pass backward to follower segment 2. The process will continue until reaching the target.

**Figure 3:**
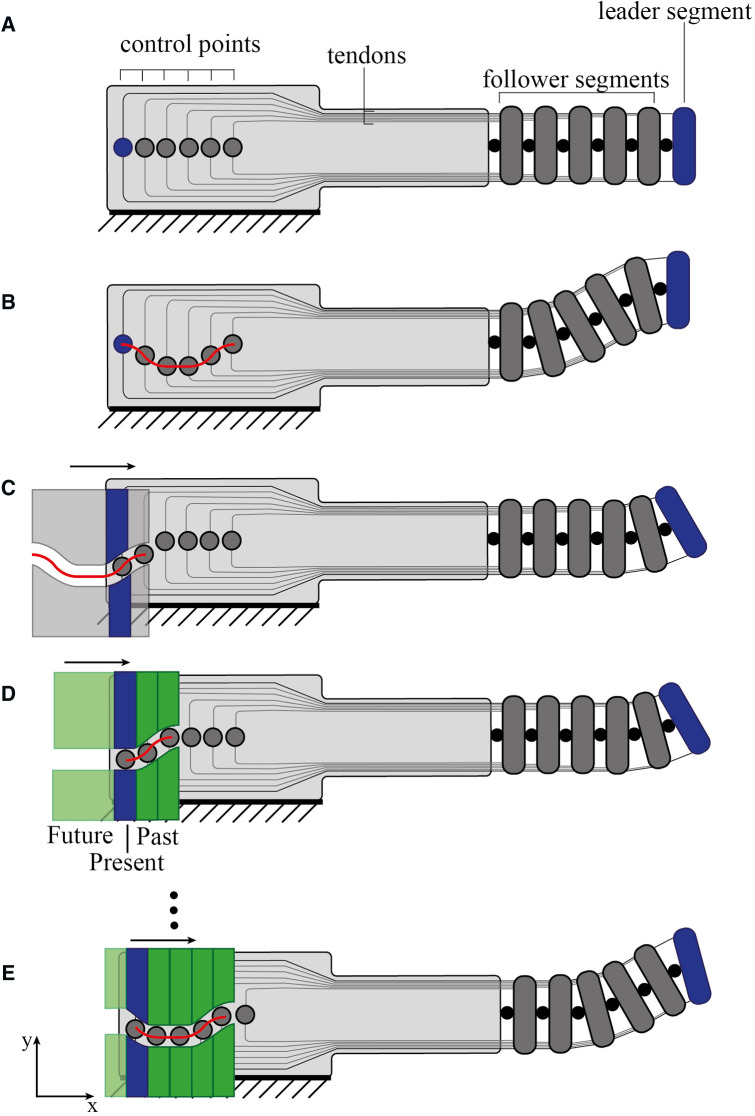
Schematic representation of a six-segmented system employing follow-the leader (FTL) propagation. (**A**) Main components of a cable-driven FTL system, showing: the control points (grey circles) with the leader control point that controls the leader segment in blue, the cables, and the follower segments. (**B**) A path is defined by individually controlling the control points. (**C**) A pre-defined physical track, superimposed to the system, can substitute the individually controlled points giving a pre-defined path. (**D,E**) The pre-defined physical track is replaced by a programmable physical track composed out of a number of steering elements that can be individually translated along the *y*-axis and locked into position. Light green represents the future, blue represents the present, and dark green the past. The red line represents the path.

Figure 3A shows a 2D representation of an FTL system with six segments. The bending angle of the leader segment (blue in Figure 3A) corresponds to a translation along the *y*-axis of the corresponding control point (also blue). As previously discussed, due to the symmetry of the system, the displacement Δs of the tensioned cable is equal to the released Δs of the antagonist cable. By steering the leader segment, memorizing its pose, and advancing the system forward along the *x*-axis, the information is passed backward. This information is the displacement of the leader control point passes to the second control point, the information of the second control point to the third, and so on (Figure 3B). The three actions, steering, memorizing, and advancing, are repeated until reaching the target area. In the representation of Figure 3B, each control point has to be independently actuated to pass back its position to the follower segment and to take the new position from the previous control point. By superimposing a pre-defined physical track on the set of control points, we pass from an FTL-mechanism that acts directly on the control points to an FTL-mechanism integrated into the pre-defined physical track. This means that, in an ideal situation, a fixed physical track is capable of taking over the role of the single actuators and perform a pre-programmed path (Figure 3C).

Using a pre-defined physical track, however, means that the track cannot be changed in real-time but can only follow the pre-defined path, which is a disadvantage (21). Discretizing the pre-defined physical track into a set of steering elements that can be translated into position and locked gives a solution (Figures 3D,E). In such a programmable physical track, the steering elements can be divided into three groups resembling the past, the present, and the future. The past (dark green) corresponds to the steering elements already translated into position and locked. The present (blue) corresponds to the steering element that will be the next one to assume a new position, and therefore the leader element that controls the leader segment of the tip. The future (light green) corresponds to the steering elements not yet defined in their position. The main functions that such a programmable memory system must provide are then:

(1) Steering the leader segment by translating the leader control point
(2) Memorizing the position given to the leader control point
(3) Advancing while passing the pose information memorized backwards to the follower control points (leader to second, second to third, etc.)

Pre-defined physical tracks can pass the pose information backward (III) but do not allow the position to be controlled in real-time (I–II).

### Memory mechanism

**Figure 4:**
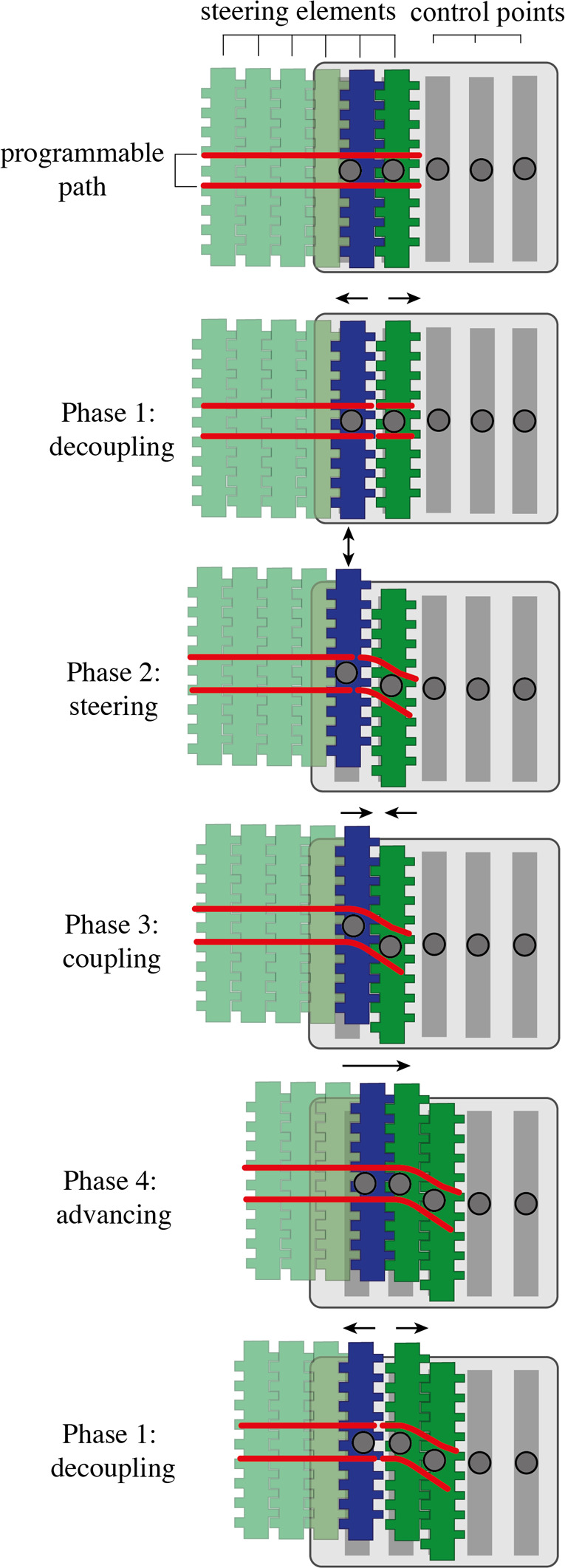
Memory mechanism showing the programmable path (red lines) and the steering elements (light green for the future, blue for the present, dark green for the past). The control points are represented by the grey dots. The figure shows the four phases required to create a real-time FTL-motion. Phase 1: decoupling the future and the present from the past. Phase 2: steering the element that represents the present to a new position. Phase 3: coupling the present and the past again to memorize the new position. Phase 4: advancing the programmable physical track along the control points to pass the pose information backward.

As shown in Figure 4, to program a path, the steering element representing the present (blue) must be decoupled from the past (Phase 1: decoupling) and moved to a position of the user's choice (Phase 2: steering). In this phase, the future steering elements (light green) are locked to the present steering element and travel with it as a single part. When the desired position is reached, the present steering element is coupled again with the past (Phase 3: coupling), the system is advanced one step forwards (Phase 4: advancing), and a new steering element becomes the blue present. This new present steering element is then again decoupled from the past, and the cycle repeats. The past (dark green) remains locked in place, and, as the cycle repeats, more steering elements are added to the past to form a programmable path, visualized by the red lines in Figure 4.

**Figure 5:**
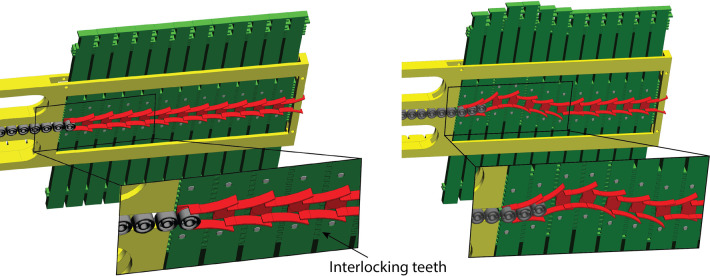
Detail of the steering element (green), guiding units (red), and control points (dark grey). At the left, the steering elements and the guiding units form a straight path. At the right, they form a curved path.

Figure 5 shows a close up of the programmable physical track in a 3D representation. The steering elements (green in Figure 5) create a discretized path by interlocking with each other due to teeth positioned at their sides. For smooth steering of the control points over the programmable physical track, we equipped the steering elements with guiding units (red in Figure 5). These guiding units have thin-walled flexible lateral flaps and interpolate the discrete information from the steering elements into a smoothly curved path to guide the control points (dark grey in Figure 5). Each guiding unit is connected to the corresponding steering element *via* a pin that enables its rotation.

## Proof of concept prototype

**Figure 6:**
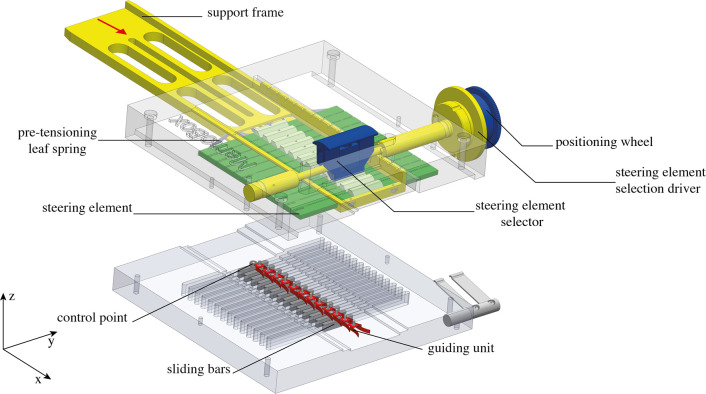
Exploded view of the MemoBox CAD model. The steering elements are shown in green and the guiding units in red. The steering elements are positioned in a support frame (yellow) that slides over the control points (dark grey ball bearings) connected to sliding bars (dark grey) along the *x*-axis. In order to determine a new position for the present steering element, the steering element selector (blue) engages the present steering element, and the positioning wheel (blue) enables its translation along *y*-axis. When the new position is achieved, the steering element selector disengages the present steering element, and the pre-tensioned leaf spring pushes back the steering elements in order to lock them in position. The programmable physical track slides over the control points by rotating the steering element selection driver (yellow) in the direction of the red arrow (*x*-axis).

A proof-of-concept MemoBox prototype was manufactured in order to test the functioning of the programmable physical track proposed in the previous section, Figure 6. The prototype was designed by keeping in mind the size of the pre-defined physical tracks of the MemoFlex II (60 mm × 30 mm × 4 mm) (21). The MemoBox mechanism is surrounded by a rectangular clear acrylic frame (light grey) that is split into two parts; the top part guiding the memory mechanism and the bottom part guiding the control points. The bottom part of the acrylic frame contains slots that guide sliding bars (dark grey) with the control points, represented by Ø3 mm ball bearings. The sliding bars represent the connection with the cables of the snake-like instrument, and they can only translate sideways. As the programmable physical track must slide over the control points to transfer the path (Figure 4) along the *x*-axis, the steering elements are mounted into a moving support frame (yellow) and coupled together by a pre-tensioned leaf spring (grey).

In order to decouple the past steering elements from the present and future steering elements, the present steering element is engaged by a steering element selector, blue in Figure 6. By pushing it manually downwards, the steering element selector creates a space between the past and the present. Unlocking the past from the present enables the translation along the *y*-axis (y-translation) of the present and future steering elements without interfering with the stored path. Once the position of the present is defined, the steering element selector disengages the present steering element, and the pre-tensioning leaf spring pushes the present back together to the past reconnecting all the steering elements. The y-translation of the present steering element is controlled by a positioning wheel that is connected to the steering element selector, blue in Figure 6, by means of an endless screw. When the steering element selector engages the present steering element, its y-translation can be set by rotating the positioning wheel in both directions with a resolution of 1 mm per step (0.5 mm tooth thickness, 0.5 mm gap between two teeth). The maximum travel range (y-translation) between adjacent segments was limited to 2 mm in either direction to avoid creating an irregular path that would be unable to guide the ball bearings (control points) smoothly. Finally, a knob named the steering element selection driver, yellow in Figure 6, can be used to move forward and backward the programmable physical track along the control points.

**Figure 7:**
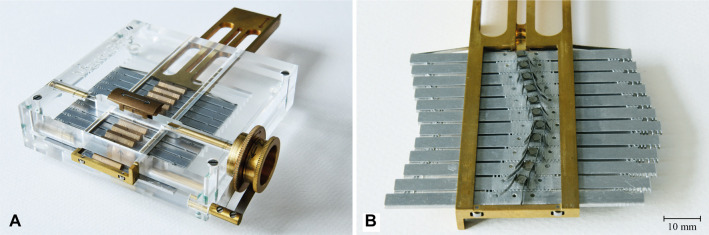
The MemoBox prototype. (**A**) Top view of the prototype; (**B**) a detail of the smooth path formed by the guiding units.

The steering elements were machined out of an aluminum plate by means of Electric Discharge Machining (EDM). Sliding parts, such as the support frame, the positioning wheel, the steering element selection driver, and the sliding bars, were made out of brass to minimize friction. All springs in the system were made out of stainless spring steel. The frame was made of clear Acrylic to facilitate visualization and analysis of the mechanism's behavior and the motion of the control points over the path (Figure 7).

## Functional evaluation

**Figure 8:**
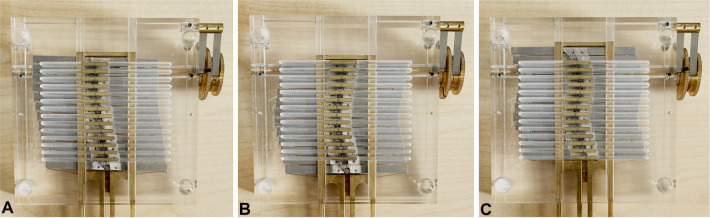
Different paths followed by MemoBox. (**A**) Single curve; (**B**) double curve; (**C**) multi curve.

The MemoBox prototype was used to test the setting mechanism of the programmable track and to evaluate its smooth propagation on the control points and their guiding units. MemoBox is able to follow a wide range of single and double or multi curved paths (Figure 8).

**Figure 9:**
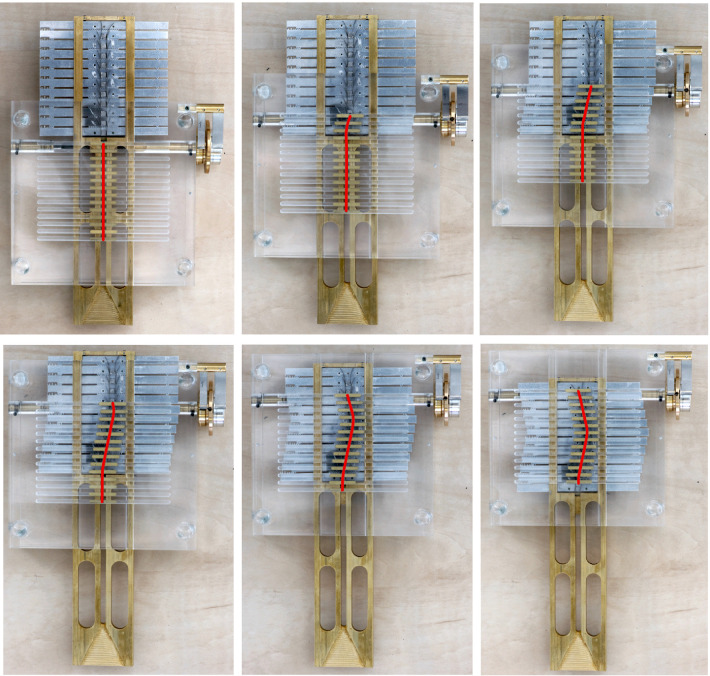
MemoBox following a triangular path, corresponding to a double curved shape in a snake-like shaft. The programmable physical track slides over the control points step-by-step, and each time the selected steering element takes the new position decided by the user.

Figure 9 shows the sequence of motions to form a triangular path; from the starting position, in which the control points are in their initial straight position and still separated from the steering elements, to the ending position, in which all the steering elements are engaged to form the path and have slid over all the control points. Considering the behavior of a hypothetical steerable shaft (as shown in Figure 2), in the beginning, the shaft will then be straight, whereas, in the end, it will take a double-curved position due to the translation of the control points. Moreover, the travel range given by the memory mechanism (i.e., the displacement of the steering cables) and the shaft diameter directly control the angle of curvature of the shaft, as shown in Figure 2. Therefore, given the maximum travel range of 2 mm, the larger will be the diameter of the shaft, the smaller will be the angle of curvature of the shaft. The action sequence of the user follows the four main phases listed in Section “Concept design”. First, the selector is pushed down to engage the present steering element and space it to the past steering elements. Once the present steering element is engaged, the new position can be selected by turning the positioning wheel clockwise or counterclockwise, depending on the direction the present steering element has to take. When the new position is decided for, the selector is released, reconnecting the present steering element with the past steering elements, and allowing the pose information to be memorized. Then, the selection driver is turned clockwise, and the next present steering element is selected, allowing the starting of a new cycle and moving the control points along the generated track. The programmable physical track works in both directions; by turning the selection driver counterclockwise, the control points follow the created path backward till they are in the neutral straight position again. This corresponds with the situation in a real surgical scenario, where the snake-like instrument will be able to follow the same path for reaching the target area and for retraction, avoiding sensitive organs or tissues on the way in and out of the patient. In the Supplementary Material of this article, a video shows the user sequence of actions and the behavior of MemoBox.

## Discussion

### MemoBox design

In this paper, we developed a new, fully mechanical, programmable physical track for controlling snake-like surgical instruments. MemoBox is based on the principle that instead of using 14 actuators to perform an FTL-motion, one for each control point, the control points can be controlled by superimposing a mechanical memory system. The new memory system is based on a discrete geometrical interlocking mechanism between 11 steering elements. The steering elements form the shape that is followed by the control points. The control points, 14 in our prototype, represent the number of segments of the shaft. Therefore, the steering elements can hypothetically change in number from the control points as they belong to two independent assemblies in the MemoBox with guiding units in-between. This mechanism is more reliable and stiffer as compared to continuous friction-based mechanisms, such as in alternating robots because it avoids error accumulation along the path. MemoBox allows for single, double or, multi-curved paths that can be adjusted at any time during the motion.

In our prototype, steering element discretization is 1 mm with a maximum travel range of ±2 mm from one steering element to the next. In combination with an Ø8 mm steerable shaft like the one in Figure 2, with the cables placed concentrically in a Ø6.4 mm ring, the shaft can reach a bending angle of 180 degrees with a cable displacement of ±10 mm. This means that, with a maximum travel range of ±2 mm, the shaft can reach an angle of 180 degrees in five subsequent steps of the mechanical memory system, with 36 degrees per step. If, instead, we consider a Ø5 mm shaft with cables placed in a Ø4 mm ring, the shaft would reach the same bending angle in three steps of the mechanical memory system. In our prototype this resolution was considered sufficient (21). However, the resolution can be further improved by decreasing the tooth width and spacing. Moreover, MemoBox is a modular system and the number of steering elements can be adapted depending on the number of DOF needed for the selected procedure. In our prototype, the 11 steering elements can each take five possible positions: two positions when the steering element translates upwards along the *y*-axis, two when it translates downwards, and one of it remains in the middle with respect to the follower segment. Therefore, there are theoretically 511 possible paths that the mechanical memory can perform.

MemoBox used guiding units with flexible stainless-steel flaps to interpolate the discretized information of the steering elements in a smooth path. However, due to limitations in the flexibility of the flaps, the travel range between two adjacent elements cannot be greater than ±2 mm in order to not create irregularities. An alternative solution to increase the travel range and thus achieve a larger bending angle with fewer steering elements in the instrument shaft, could be the implementation of a continuous compliant element connected to the steering elements to create a smooth and continuous path.

Although the user of the memory system has a continuous path in mind, our MemoBox requires translation of this continuous motion into a discrete input. At each step, the user selects the present steering element, defines the new position, and moves the memory one step forward. Even though the control points move smoothly from one position to another, the sequence of motion set by the user remains discretized. The discretization of the FTL movement is, however, an intrinsic characteristic of FTL surgical robotic systems, as the pathway is always programmed in a step-wise manner related to the number and length of the segments.

MemoBox represents only one module of the overall system, and therefore a complete evaluation of the FTL error cannot be carried out here. However, one of the main factors that influence the FTL error in our MemoBox is the discretized angle resulting from the resolution in the translation of the steering elements. The 1 mm resolution allows the segments of the previously considered Ø8 mm compliant shaft to make discrete steps at an interval of 18 degrees. Therefore, considering a segment length of 10 mm, the maximum discretization error would be around 1.5 mm, given by half of the 18 degrees angle at 1 mm discretization multiplied by the length of the steerable segment. The acceptable error mainly depends on the surgical application. An error of 1.5 mm is comparable with the error of similar FTL systems (27, 38) and, although still too large for sinus surgery (39), is tolerated in procedures such as colonoscopy (12). Scaling down the resolution of the steering elements would reduce the discretization error and expand the application range. Besides the discretization, FTL behavior would also be affected by friction, flexible behavior of the steering cables, and a possibility of cable buckling. All those factors should be considered beforehand when designing the overall system to predict possible discrepancies from the estimated path and guarantee the safety of the system (21).

Besides MemoBox, the literature contains only one other mechanically programmable physical track called MemoSlide (40). One of the difficulties encountered in MemoSlide was miniaturization. In MemoBox, greater miniaturization has been achieved by replacing MemoSlide's complex alternating mechanism to memorize the path by a much simpler shape shifting mechanism. By using a shape-shifting mechanism, the number of components halved because the mechanism is based on only one memory system. Moreover, MemoSlides uses wedges that create a discretized path. In MemoBox, the flexible flaps of the guiding units replace the wedges and ensure a smooth path for the ball bearings. We, therefore, decreased the size of the programmable physical track from 145 mm × 125 mm × 25 mm of the MemoSlide to 70 mm × 64 mm × 6 mm of our MemoBox with a reduction in edge length of 50% by keeping the same discretization and the same number of programmable segments.

### Implementation in a surgical instrument

**Figure 10:**
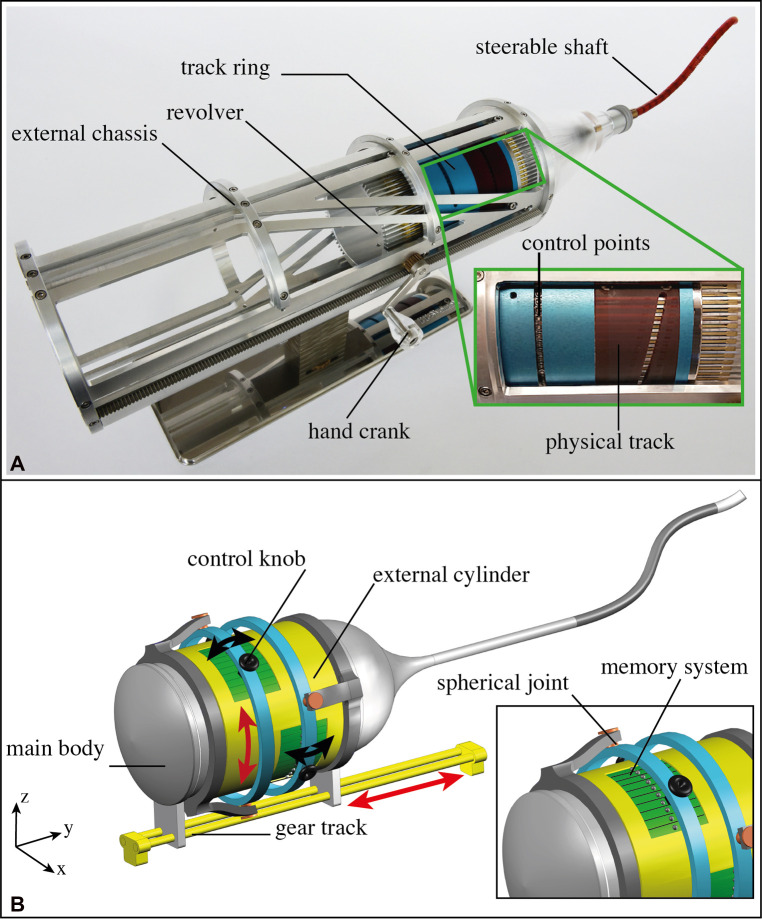
Integration of the MemoBox system into a surgical instrument with an FTL-motion. (**A**) MemoFlex II device (21). The close-up shows a physical track and the control points. (**B**) An artistic impression of the final system integrated with four MemoBox programmable physical tracks. The main body (grey) slides over a gear track (yellow). The sliding motion of the main body is coupled with the rotational motion of the external cylinder (yellow) in which the programmable physical tracks are placed (green). At each step, a new position of the present steering element is decided by using the two double-joystick, one for each plane (black). The control knobs are attached to the main body by means of support with two spherical joints (orange in the close-up).

MemoBox was designed as a programmable physical track able to guide a surgical shaft along tortuous paths with an FTL-motion avoiding sensitive organs or tissues. Being a proof of concept, we designed a 2D system able to control cables of the steerable shaft in one plane. However, in a real scenario, the steerable shaft needs to move in 3D. The integration of MemoBox into a system such as MemoFlex II (21) allows transforming a system based on pre-defined physical tracks into one that can be controlled in real-time, giving the possibility of adjusting the path during the surgical procedure. MemoFlex II is based on the combined work of four physical tracks, two for each plane, Figure 10A. Each pair of physical tracks control two antagonistic cables for each segment of the steerable shaft. The four physical tracks are placed in an external cylinder that rotates around the main body of the instrument. The external cylinder is coupled with an external chassis to combine the steering motion with the forward and backward motion of the instrument. Substituting the four pre-defined physical tracks with the MemoBox mechanism will result in the instrument schematically shown in Figure 10B. The figure shows a side view of the instrument where only two of the integrated MemoBoxes are visible, one for the motions in the xz-plane and the other for the motions on the yz-plane. The four MemoBoxes in this schematic mechanism are controlled by means of two pivotable rings, light blue in Figure 10B. The use of these rings allows the user to control antagonist MemoBoxes in a synchronized fashion. The rings are connected to the main body of the instrument *via* a frame, grey in Figure 10B, each with two spherical joints, depicted in orange. Control knobs are connected to the rings to select and control the position of the present steering elements. Following the design of the MemoFlex II, the rotational motion of the yellow external cylinder that houses the four MemoBoxes can be coupled with the sliding motion, forward and backward, in the *z*-axis. The coupling can be done similarly as in the MemoFlex II, with an external chassis, but for the sake of clarity, this external chassis is not shown in Figure 10B. MemoBox was prototyped as a first proof-of-concept and was therefore designed as a 2D planar design. Future work will focus on integrating the MemoBox into a 3D system, thereby reshaping the mechanism into a curved design.

### Strengths and limitations of mechanical over mechatronics solutions

When comparing mechatronic and fully mechanical FTL solutions, one of the advantages of using mechatronics is that each DOF is individually controlled by a dedicated actuator. Having independent control over each DOF generally makes such a system more versatile, e.g., not only suited to propagate tracks initiated by the end-effector but also suited for changing the entire track at one time. A limitation of mechatronics FTL-systems as compared to mechanical ones is the associated high complexity and related costs (41). For example, to control a snake-like tip with 16 segments, each having 2 DOF, with each of the four steering cables connected to a motor, 64 electric motors need to be precisely synchronized, as compared to only four MemoBoxes. Using 64 motors, including sensors, gearboxes, and controls, will greatly increase complexity and costs as well as the overall size of the system. In an attempt to reduce the system size, miniature electric motors can be used as an alternative. However, miniature motors can deliver only limited power, which would result, with the use of miniature gearboxes, in slow responsiveness of the system. Reducing the number of motors by making mechanical connections between antagonistic steering cables would lead to 30 motors and 30 mechanical linkages or pulleys, which will still lead to a very complex system as compared to our approach with four MemoBoxes. Therefore, mechatronic and mechanical solutions should be considered complementary and chosen depending on the specific procedure. When mechanical solutions are not able to ensure the precision and versatility requested, mechatronics systems provide a valuable solution that justifies the costs.

## Conclusion

In this work, we presented a new programmable, mechanically-actuated physical track to guide a steerable shaft through tortuous paths with FTL-motion. A 2D proof of concept prototype, called MemoBox, was manufactured able to memorize the pathways that are selected in real-time by the user and transform these pathways into the input of a snake-like multi-steerable shaft. Mechanical FTL solutions provide advantages over mechatronics solutions in controlling a high number of degrees of freedom, whereas mechatronics solutions can guarantee more versatility and precision. Mechanical and mechatronics FTL solutions should be therefore considered complementary and chosen depending on the specific surgical procedure. FTL-instrumentation can make a difference in many surgical scenarios, such as colonoscopy, interventional bronchoscopy, or skull base surgery. MemoBox represents a step forward in designing advanced snake-like surgical instruments without the use of actuators and electronics components.

## Data Availability

The original contributions presented in the study are included in the article/**Supplementary Material**, further inquiries can be directed to the corresponding author/s.
